# Complete Nucleotide Sequence of an Isolate of Grapevine Satellite Virus and Evidence for the Presence of Multimeric Forms in an Infected Grapevine

**DOI:** 10.1128/genomeA.01703-16

**Published:** 2017-04-20

**Authors:** T. Candresse, A. Marais, S. Theil, C. Faure, T. Lacombe, J. M. Boursiquot

**Affiliations:** a UMR 1332 Biologie du Fruit et Pathologie, INRA, Université Bordeaux, Villenave d’Ornon, France; b UMR 1334 AGAP, INRA, Montpellier SupAgro, Montpellier, France; c Centre de Ressources Biologiques de la Vigne, INRA, Marseillan-Plage, France

## Abstract

The complete nucleotide sequence of an isolate of grapevine satellite virus (GV-Sat) was determined by next-generation sequencing (NGS) and compared with the single available complete sequence. The NGS data unexpectedly provided evidence for the existence of multimeric forms of GV-Sat, which were experimentally confirmed, allowing the redefinition of GV-Sat genomic ends.

## GENOME ANNOUNCEMENT

Grapevine satellite virus (GV-Sat) is a recently discovered agent with a reported prevalence of about 3% in two grapevine collections in the United States (1). Its genome (KC149510) harbors two open reading frames (ORFs) that may be translationally coupled through frameshifting. The largest protein shows weak homologies (1) with the P20 of *Bamboo mosaic virus satellite RNA* (2) and with the capsid protein of *St. Augustine decline satellite virus* (3) and *Panicum mosaic satellite virus* (4).

Double-stranded RNAs from grapevine cv. Askeri in Iran held in the collection of INRA (Domaine de Vassal, Marseillan, France) were purified, randomly amplified, and Illumina sequenced according to Candresse et al. (5). After quality trimming, contigs were assembled and annotated by BlastX and BlastN comparison with GenBank using the CLC Genomics Workbench 8 and in-house developed pipelines. A single large GV-Sat scaffold was obtained from 229 reads (0.8% of the total reads), providing a 47× genome coverage. Remarkably, sequencing reads were identified spanning the junction between the 5′ and 3′ genomic ends, suggesting the existence of multimeric forms of GV-Sat. This was verified in RT-PCR experiments employing the GV-Sat 1F and GV-Sat 1R divergent primer pair (5′ GGTAAACTTCGTCGATGACGA 3′ [nucleotides (nt) 659 to 679] and 5′ AATGCTGTTTGCGTCCCTGCG 3′ [nt 278 to 258], respectively) which cannot amplify a product from the monomeric GV-Sat but yielded the 668 bp product expected from an amplification of multimeric GV-Sat. Sequencing the amplicon validated the sequence predicted from the NGS reads, linking the 3′ and 5′ genome ends. Partial sequencing of a second GV-Sat isolate from a different grapevine also provided similar evidence, confirming the existence of GV-Sat multimers. Such multimers have been previously reported for several linear satellites (6, 7) and for a genomic RNA of several tospoviruses (8) but not so far in a satellite virus.

As determined from its multimeric form, the genome of GV-Sat Askeri is 1048 nt long (KY211673). It is essentially colinear with KC149510 in the coding region, with a single 6 nt insertion near the ORF1 start codon. The overall nucleotide identity between the two isolates is 92.8%. The ORF1 protein shows 90.7% amino acid identity, the ORF2 98.9%. Indel polymorphisms are observed in the 5′ and 3′ noncoding regions, in particular, a large 22 nt indel near the 3′ genome end. The most striking difference concerns the interpretation of the genome ends. Indeed, the sequence 5′GTATTGTGCTA3′ at the extreme 3′ end of the genome is also found near the 5′ genome end in KC149510 suggesting that the RACE experiments performed during the initial characterization of GV-Sat may have misinterpreted the sequencing of a multimer. Analysis of multiple reads spanning the junction and of nonviral nucleotides found in that junction in some of the reads (6, 7) indicates that the correct genome ends correspond to nucleotides 32 and 1,049 of KC149510. In conclusion, the sequence reported here shows the existence of significant sequence variation in this agent and the demonstration of the existence of multimeric forms of GV-Sat in infected grapevines allows the proposal of revised genome ends of this poorly characterized virus.

### Accession number(s).

The complete genomic sequence of GV-Sat reported here has been deposited in GenBank under accession number KY211673.
